# Central Adiposity Indicators by Pre-Pregnancy BMI at the 23 kg/m^2^ Cut-Off in Two Independent Cohorts of Thai Women During Pregnancy and Postpartum: A Descriptive Longitudinal Study

**DOI:** 10.3390/nursrep16080263

**Published:** 2026-07-29

**Authors:** Piyanut Xuto, Piyaporn Prasitwattanaseree, Lawitra Khiaokham, Daniel Bressington, Patompong Khaw-on

**Affiliations:** Faculty of Nursing, Chiang Mai University, 110/406 Intawaroros Rd., Suthep, Muang, Chiang Mai 50200, Thailand; piyaporn.p@cmu.ac.th (P.P.); prueksalada.k@cmu.ac.th (L.K.); daniel.bressington@cmu.ac.th (D.B.); patompong.kh@cmu.ac.th (P.K.-o.)

**Keywords:** nursing, body mass index, maternal obesity, postpartum weight retention, waist-to-height ratio, Asian populations, Thai women

## Abstract

**Objectives:** Prospective anthropometric data on how women above and below the Asian-specific BMI ≥ 23 kg/m^2^ cut-off differ across the maternal continuum remain limited. Because these data were collected in 2017–2018, before Thailand’s 2022 adoption of the threshold in antenatal care, this study describes central adiposity indicators in two independent cohorts of Thai women as a pre-implementation baseline. **Methods**: In this descriptive longitudinal dual-cohort study at a tertiary centre in Northern Thailand, 192 women formed two independent cohorts: Cohort A (pregnancy, from 12 weeks’ gestation; *n* = 110) and Cohort B (delivery to 24 weeks postpartum; *n* = 82), with no woman in both. Women were stratified by pre-pregnancy BMI (<23 vs. ≥23 kg/m^2^). Waist-to-height ratio (WHtR, primary) and mid-upper arm circumference (MUAC, secondary) were measured at seven timepoints and validated against 3D body scanning (r = 0.92–0.99). **Results**: At 12 weeks’ gestation, the ≥23 kg/m^2^ stratum had higher WHtR (0.58 vs. 0.49; d = 1.56) and MUAC (32.52 vs. 26.19 cm; d = 1.98; both *p* < 0.001). Postpartum, the <23 kg/m^2^ stratum returned to WHtR < 0.50 by Week 8, whereas the ≥23 kg/m^2^ stratum remained above 0.50 through 24 weeks (0.56 vs. 0.48; *p* < 0.001). **Conclusions**: The ≥23 kg/m^2^ stratum showed higher central adiposity indicators from early pregnancy and, in the separate postpartum cohort, values that remained above 0.50 through 24 weeks. Because the cohorts comprised different women, these patterns are descriptive and cannot demonstrate within-woman retention. They nonetheless suggest the cut-off may identify women who could benefit from earlier antenatal and extended postpartum nursing assessment, pending confirmation in within-woman studies.

## 1. Introduction

The rising global prevalence of obesity poses increasingly serious consequences for maternal and reproductive health [1], yet the tools used to screen pregnant and postpartum women for metabolic risk have not kept pace with accumulating evidence on ethnic variation in body composition [2,3]. The conventional World Health Organization (WHO) overweight threshold of BMI ≥ 25 kg/m^2^—derived primarily from Caucasian population data—fails to capture the disproportionate metabolic vulnerability observed in Asian individuals at lower body weights [2]. This limitation underpins the clinically documented Asian paradox or thin-fat phenotype: elevated cardiometabolic dysfunction at BMI levels that standard criteria classify as normal weight [4,5].

A physiological basis for this pattern has been described. Asian populations accumulate visceral adipose tissue preferentially at lower absolute body weights compared with European populations [6], and visceral fat exerts greater lipolytic activity, inflammatory cytokine secretion, and insulin-sensitizing disruption than subcutaneous depots [7]. Recognizing this, the WHO Expert Consultation recommended lowering the overweight cut-point for Asian populations to BMI ≥ 23 kg/m^2^ and the obesity threshold to ≥27.5 kg/m^2^, with explicit acknowledgement that revised criteria better identify individuals at increased metabolic risk [2,3]. A Southeast Asian obesity consensus—representing 10 countries (Bangladesh, Brunei Darussalam, India, Indonesia, Malaysia, Philippines, Singapore, Sri Lanka, Thailand, and Viet Nam)—has endorsed this cut-point as the appropriate clinical action point for intervention [8].

Despite this convergent international guidance, adoption of the Asian-specific BMI threshold in maternal health settings remains inconsistent. In Thailand, the Department of Health, Ministry of Public Health (MoPH) has more recently endorsed BMI ≥ 23 kg/m^2^ as the overweight threshold in its 2022 national antenatal care manual, recommending additional nutritional counselling for women above this level [9]. This national guidance, however, postdates the 2017–2018 period in which the data analyzed here were originally collected (see Methods); at the time of data collection the ≥23 kg/m^2^ cut-off was available internationally through the 2004 WHO Asian criteria but was not yet embedded in a Thai antenatal directive. Because the present data predate the 2022 national recommendation, this secondary analysis is best understood not as a contribution to guideline implementation but as a retrospective description of the pre-implementation period—an anthropometric baseline against which the impact of the current recommendation can be evaluated in future work. Even under this recommendation, however, uptake of the lower cut-off into routine antenatal nursing assessment is likely to be variable, and the anthropometric evidence base for applying it across the maternal continuum remains limited. We therefore frame incomplete implementation as a plausible practice gap rather than an established finding. Women in the BMI 23–24.9 kg/m^2^ range—classified as normal weight by the traditional threshold—present routinely to antenatal clinics across the region without triggering screening or counselling pathways. Healthcare providers, including nurses who are the primary point of contact for antenatal and postpartum assessment, face uncertainty regarding which women warrant early nutritional intervention. This ambiguity is consequential: excessive gestational weight gain strongly predicts metabolic complications such as gestational diabetes in the index pregnancy [10], while inadequate postpartum weight loss drives long-term obesity and cardiovascular risk [11]. Together, these compounded factors elevate the risk of hypertensive complications and metabolic disease in subsequent pregnancies and in offspring [1,12].

Despite these epidemiological signals, prospective anthropometric data describing how women above and below the BMI 23 kg/m^2^ cut-off point differ across the maternal continuum remain limited. While prior work from Singapore [13] and India [4] incorporated advanced body composition tools like DXA and bioelectrical impedance, these studies were cross-sectional and not specific to the physiological changes of pregnancy. Conversely, prospective longitudinal studies in maternal settings, such as those in Thailand [12], have relied on BMI alone. Two specific questions remain unanswered: first, whether women above this threshold already elevate visceral adiposity in early pregnancy—before significant gestational weight gain—and second, whether that adiposity persists postpartum beyond the window of physiological recovery.

Waist-to-height ratio (WHtR) addresses a persistent methodological concern in maternal anthropometry by normalizing waist circumference for height, with a threshold of 0.50 widely recognized as indicating elevated cardiometabolic risk across multiple ethnic groups [14,15,16]. WHtR is associated with higher glucose concentrations in early pregnancy [17] and is suitable for serial measurement without specialist equipment. Mid-upper arm circumference (MUAC) provides an independent peripheral adiposity marker unaffected by abdominal pregnancy-related changes, enabling validation of central measurements during gestation [18,19].

These constructs are distinct and are used here with care. BMI is a whole-body mass index and, in this study, serves only to define the strata; WHtR and MUAC are anthropometric indicators of central and peripheral fat distribution, respectively; visceral adiposity refers to the specific intra-abdominal fat depot, which can be quantified only by imaging; and metabolic risk is a physiological state confirmed only by biomarkers. This study measured the anthropometric indicators (WHtR, MUAC) and treats them throughout as surrogates of central adiposity, not as direct measures of visceral fat or of metabolic risk.

The present study addressed this evidence gap through a prospective design comparing two independent cohorts of different women across the maternal continuum. We examined two descriptive hypotheses: that women with pre-pregnancy BMI ≥ 23 kg/m^2^ would show (a) higher central adiposity indicators—a pattern we refer to descriptively as hidden obesity—detectable at 12 weeks’ gestation, when the fetal contribution to abdominal girth is negligible, and (b), in a separate postpartum cohort, higher and more slowly declining WHtR—a pattern we refer to descriptively as sticky-fat—through 24 weeks postpartum, beyond the period of uterine involution. Because the two cohorts comprise different women, these patterns represent group-level anthropometric differences rather than demonstrated within-woman fat retention. Consistent findings would provide preliminary anthropometric evidence to inform, rather than by themselves justify, discussion of maternal nursing assessment across Asian health systems.

## 2. Materials and Methods

### 2.1. Study Design and Setting

This prospective longitudinal study employed a dual-cohort design to examine anthropometric patterns across the maternal continuum. Critically, the two cohorts comprised different women: Cohort A (pregnancy) and Cohort B (postpartum) were recruited as separate samples, and no participant contributed to both. The design, therefore, compares independent groups at different stages of the maternal continuum and does not follow the same women from pregnancy into the postpartum period. The investigation involved a secondary analysis of data collected between October 2017 and August 2018 at Maharaj Nakorn Chiang Mai Hospital, a tertiary referral center in Northern Thailand. Both cohorts were derived from the Z-Size Ladies project, a parent study of body shape patterns in Thai women using validated three-dimensional body scanning technology [20]. The parent study established standardized anthropometric measurement protocols and validated self-measurement techniques against reference scanner data, achieving correlation coefficients exceeding 0.90 for all key measurements. This study adheres to the STROBE guidelines for reporting observational studies [21]; a completed STROBE checklist is provided as Supplementary Materials.

Relationship to the parent study. Both cohorts are drawn from the Z-Size Ladies parent project, whose primary output was a three-dimensional body-shape simulation study [20]. The present secondary analysis uses the same underlying anthropometric dataset, and the apparent differences in participant numbers between the two papers reflect different denominators rather than different data. The parent paper characterised the pregnancy sample by the number of women measured at a single gestational timepoint, whereas the present analysis counts all eligible women who contributed at least one measurement (*n* = 110); the per-timepoint counts in the two papers are identical. For the postpartum cohort, the present analysis excludes one woman (*n* = 82 vs. 83) who lacked the documented pre-pregnancy weight required for BMI stratification, a variable not needed for the parent paper’s simulation equations. The accuracy-testing volunteers reported in the parent paper formed a separate validation sample and contributed no longitudinal data; they are not part of either cohort analysed here.

### 2.2. Cohort Design and Timepoints

Cohort A—Pregnancy cohort (*n* = 110) was established to test the hidden obesity hypothesis through serial anthropometric assessment across seven gestational timepoints: 12, 16, 20, 24, 28, 32, and 36 weeks of gestation. These serial timepoints were not selected at random; they correspond to the routine four-weekly antenatal visit schedule at the study site, allowing measurements to be obtained during scheduled clinical contacts without additional participant burden. The 12-week assessment served as the primary baseline for between-group comparisons because at this time point, the fetal crown-rump length measures between 45 and 84 mm [22]. The uterus has also just begun to emerge from the pelvis, minimizing confounding from fetal mass and amniotic fluid volume. Repeated measurements through 36 weeks enabled characterization of longitudinal anthropometric trajectory patterns throughout the second and third trimesters.

Cohort B—Postpartum cohort (*n* = 82) was designed to test the sticky-fat hypothesis by tracking weight retention trajectories. Participants were recruited within 72 h of delivery and assessed prospectively in weeks 0, 4, 8, 12, 16, 20, and 24 postpartum. These four-weekly intervals were pre-specified to capture uterine involution and the early postpartum weight-change trajectory. This follow-up duration extends beyond the 6–8-week period of uterine involution, ensuring that persistent anthropometric differences reflect adipose tissue retention rather than incomplete physiological recovery.

### 2.3. Participants

Eligible participants were Thai women aged 18–45 years receiving prenatal or postpartum care at the study site. Self-identified Thai ethnicity was confirmed at enrolment. Inclusion criteria required a singleton pregnancy and the ability to provide written informed consent. Exclusion criteria included known fetal anomaly, pre-existing diabetes mellitus or hypertensive disorder, use of medications affecting body composition (corticosteroids or antipsychotics), and physical disability preventing accurate anthropometric measurement.

Participants in both cohorts were stratified by true pre-pregnancy BMI, calculated from the pre-pregnancy weight and height documented in the antenatal medical record (weight in kilograms divided by height in metres squared); early-pregnancy BMI was not used for stratification in either cohort into two strata: a Low-Risk stratum (BMI < 23 kg/m^2^, normal weight by the WHO Asian criteria) and an At-Risk stratum (BMI ≥ 23 kg/m^2^, overweight or obese by the same criteria). These labels denote position relative to the BMI cut-off under examination and are not independently validated risk categories. Because the parent data were collected in 2017–2018, this ≥23 kg/m^2^ stratification was applied retrospectively for the present secondary analysis, using the internationally available 2004 WHO Asian cut-off rather than any protocol in force at the study site at that time. Dichotomization at this single cut-off—rather than multiple BMI subgroups—was chosen to examine the ability of the cut-off to distinguish anthropometric patterns while preserving statistical power and interpretability.

### 2.4. Anthropometric Measurements

All measurements were obtained by trained research nurses following standardized protocols established in the parent study [20]. Participants wore light clothing without shoes and voided their bladder immediately before measurement. Measurements were obtained in a private examination room at 22–24 °C ambient temperature.

Height was measured using a wall-mounted stadiometer (Seca 206, Hamburg, Germany) to 0.1 cm precision with participants in the Frankfort horizontal plane. Weight was measured using a calibrated digital scale (Tanita BC-418, Tokyo, Japan) to 0.1 kg precision; pre-pregnancy weight was obtained from the antenatal medical record for all participants in both cohorts; no self-reported weights were used (0% self-report). BMI was calculated as weight in kilograms divided by height in meters squared.

For clarity of definition, waist-to-height ratio (WHtR) was computed for each woman as waist circumference divided by height, and mid-upper arm circumference (MUAC) denotes the right upper-arm circumference throughout (the left arm was also recorded and yielded materially identical results). All between-stratum comparisons at fixed timepoints used Welch’s *t*-test (which does not assume equal variances), with Cohen’s d calculated from the pooled standard deviation. Because the number of women contributing a measurement varies by timepoint, all descriptive statistics, test statistics, effect sizes, and confidence intervals are computed on the women with a valid measurement at the timepoint concerned, and the corresponding n is reported for every timepoint in Supplementary Tables S1–S4.

Hip circumference (maximum posterior extension of buttocks), chest circumference (fourth intercostal space at mid-respiration), and right leg circumference (mid-thigh midpoint) were measured using standardized protocols from the parent study [20]; full protocols are reported elsewhere. All measurements were obtained in triplicate and repeated at each serial assessment timepoint.

### 2.5. Data Quality Assurance

Measurement validity was confirmed against the Size Stream SS20 three-dimensional body scanner in a subset of participants (*n* = 45), yielding excellent concurrent validity for height (r = 0.99), waist circumference (r = 0.92), and MUAC (r = 0.94). Self-measurement reliability was confirmed in a further subset (*n* = 30; ICC > 0.95 for all sites).

### 2.6. Statistical Analysis

Data were analyzed using SPSS Version 20.0 (IBM Corporation, Armonk, NY, USA). Between-group differences at each pre-specified timepoint were assessed using independent-sample *t*-tests; effect sizes were calculated as Cohen’s d. Statistical significance was set at *p* < 0.05 (two-tailed). The study employed two pre-registered primary outcomes (WHtR at 12 weeks’ gestation for Cohort A; WHtR at 24 weeks postpartum for Cohort B), consistent with a confirmatory hypothesis-testing framework rather than exploratory multiple testing; accordingly, Bonferroni correction was not applied to these pre-specified primary comparisons, and *p*-values at serial timepoints are reported descriptively to characterize trajectory patterns. Because the dual-cohort design recruited independent participant groups rather than following a single longitudinal panel with repeated within-person measurements, repeated-measures mixed-effects modelling did not apply to the primary between-group comparisons. Because each cohort contributes repeated measurements on the same women, within-cohort longitudinal data were additionally analysed using linear mixed-effects models (random intercept per participant, maximum-likelihood estimation), fitted separately in each cohort for WHtR and MUAC. Models included fixed effects for BMI stratum (group), timepoint (time), and their interaction (group × time), allowing formal tests of between-stratum differences, change over time, and whether trajectory shape differed between strata. Mixed-effects models use all available observations rather than complete cases only, providing valid inference under a missing-at-random assumption. In addition, an attrition analysis compared the baseline characteristics of postpartum completers and non-completers. Between-stratum comparisons at individual timepoints used the Mann–Whitney U test given the small strata; full per-timepoint means, standard deviations, sample sizes, and *p*-values are reported in Supplementary Tables S1–S4, and model results in Table S5. Trajectories were visualized using Python 3 (Python Software Foundation, Wilmington, DE, USA) on Google Colaboratory (Google LLC, Mountain View, CA, USA; https://colab.research.google.com); analysis code is available upon reasonable request.

### 2.7. Ethical Considerations 

The study was conducted in accordance with the Declaration of Helsinki. The original data collection was approved by the Institutional Review Board of the Faculty of Nursing, Chiang Mai University (Code: FULL055-2017, approved 6 October 2017), and the Faculty of Medicine, Chiang Mai University (Code: NONE-2017-05134, approved 6 October 2017). All parent-study participants provided written informed consent after a full explanation of the study aims and procedures. For the present secondary analysis, which used only fully anonymized existing records and involved no new participant contact, the Research Ethics Committee of the Faculty of Nursing, Chiang Mai University, granted exempt status (Code: 2569-EXEM001; approved 19 February 2026).

## 3. Results

### 3.1. Participant Flow and Recruitment

For the pregnancy cohort, 126 women were assessed for eligibility; 16 were excluded (3 did not meet inclusion criteria; 13 declined participation), yielding 110 eligible participants who form the pregnancy cohort (Cohort A). For the postpartum cohort, 132 women were assessed; 50 were excluded before enrolment (5 did not meet inclusion criteria; 45 declined participation), yielding 82 participants with valid baseline measurements at Week 0. Of these, 57 had complete data at 24 weeks (attrition = 25 [30.5%]). Table 1 presents baseline characteristics for all 82 enrolled postpartum participants; Table 2, Part B, presents 24-week outcomes for the 57 participants who completed follow-up. Participant flow numbers are reported above; the complete STROBE flow diagram is provided as Figure S1 (Supplementary Materials).

### 3.2. Demographic Characteristics and Baseline Homogeneity

Demographic characteristics of both cohorts are presented in Table 1. In the pregnancy cohort, maternal age did not differ significantly between the Low-Risk and At-Risk groups (t = 0.80, *p* = 0.428), nor did height (t = −1.74, *p* = 0.085), confirming comparable life stages and physiological milieus. The postpartum cohort demonstrated even stronger demographic matching: age was virtually identical across groups (t = −0.06, *p* = 0.950), and height differences remained non-significant (t = −1.26, *p* = 0.213). As expected by design, pre-pregnancy BMI differed markedly between strata in both cohorts (pregnancy cohort: 20.00 ± 1.88 vs. 27.25 ± 5.47 kg/m^2^, t = −6.76, *p* < 0.001; postpartum cohort: 20.16 ± 1.80 vs. 26.63 ± 3.43 kg/m^2^, t = −9.96, *p* < 0.001).

### 3.3. Central Adiposity at 12 Weeks’ Gestation (Cohort A)

Anthropometric assessment at 12 weeks of gestation was consistent with the hypothesized pattern of greater central adiposity in the At-Risk stratum (Table 2, Part A). The At-Risk group showed larger body dimensions across all measured parameters compared with the Low-Risk group. These comparisons are based on the women who contributed a valid 12-week measurement (Low-Risk n = 83; At-Risk n = 27). Waist circumference was 14.61 cm larger (95% CI [9.81, 19.41]; t = −6.20; *p* < 0.001; d = 1.79). Hip circumference was 12.99 cm larger (95% CI [8.86, 17.12]; t = −6.42; *p* < 0.001; d = 1.94). MUAC—a marker of systemic fat stores independent of the gravid uterus—showed a mean difference of 6.33 cm (95% CI [4.36, 8.29]; t = −6.58; *p* < 0.001; d = 1.98). The primary outcome, WHtR, was significantly higher in the At-Risk group (0.58 vs. 0.49; mean difference = 0.09; 95% CI [0.05, 0.12]; t = −5.20; *p* < 0.001; d = 1.56). All effect sizes were large. Notably, the Low-Risk mean WHtR at 12 weeks (0.49) lay below the 0.50 reference level, although it did not differ significantly from it (one-sample *p* = 0.13).

Longitudinal trajectories from 12 to 36 weeks of gestation (Figure S2, Supplementary Materials) revealed a parallel-track pattern: hip, leg, and arm circumferences maintained equivalent separation between groups throughout pregnancy, indicating that the At-Risk cohort preserved its systemic adiposity disadvantage. Waist circumference trajectories diverged progressively, suggesting preferential accumulation of central adiposity in the high-BMI group as pregnancy advanced.

### 3.4. Postpartum Anthropometric Trajectories (Cohort B)

**Figure 1:**
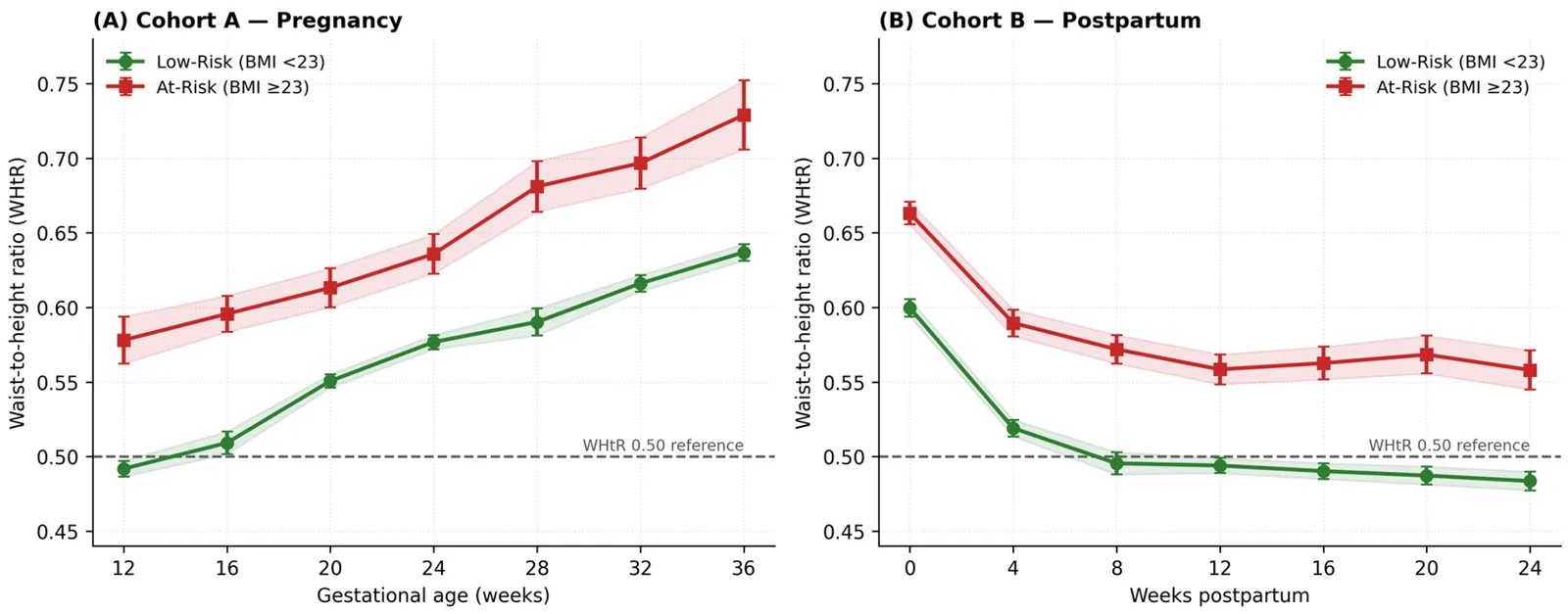
Waist-to-height ratio (WHtR) trajectories by pre-pregnancy BMI stratum in the two independent cohorts. (**A**) Cohort A (pregnancy; Weeks 12, 16, 20, 24, 28, 32, and 36 of gestation): WHtR rises across gestation in both strata. (**B**) Cohort B (postpartum; Weeks 0, 4, 8, 12, 16, 20, and 24): WHtR falls steeply to about Week 8 and then plateaus in both strata. Low-Risk: BMI < 23 kg/m^2^; At-Risk: BMI ≥ 23 kg/m^2^. Points are means; error bars and shaded bands are ±1 standard error; the number of women contributing at each timepoint varies and is reported in Supplementary Tables S1 and S2. The dashed horizontal line marks WHtR = 0.50, the level commonly associated with elevated cardiometabolic risk. In linear mixed-effects models the group and time main effects were significant in both cohorts (all *p* < 0.001), whereas the group × time interaction was not (Table S5): the At-Risk stratum remained persistently higher, with trajectories broadly parallel to the Low-Risk stratum rather than diverging. By 24 weeks postpartum the Low-Risk mean had fallen below the 0.50 level (0.484) while the At-Risk mean remained above it (0.558).

Longitudinal tracking through 24 weeks showed distinct postpartum trajectories that remained at persistently different levels rather than diverging over time (Figure 1B; group × time interaction non-significant, see below). At delivery (Week 0), both groups exhibited elevated WHtR attributable to pregnancy-related abdominal expansion; the At-Risk group began at a higher baseline (0.66 ± 0.04 vs. 0.60 ± 0.04; *p* < 0.001).

The Low-Risk group demonstrated rapid and complete recovery. WHtR declined progressively through the first 8 weeks, reaching 0.49 ± 0.05 at Week 8—falling below the 0.50 metabolic risk threshold—coinciding with the completion of uterine involution and resolution of pregnancy-related fluid retention. Values remained stable in the safe zone through Week 24 (0.48 ± 0.04).

The At-Risk group, in contrast, showed a slower and incomplete decline over the same period. WHtR declined from 0.66 ± 0.04 at delivery to 0.57 ± 0.05 at Week 8, whereupon the trajectory plateaued—a plateau in postpartum central adiposity also reported in other longitudinal cohorts—with values remaining above 0.56 through Week 24 (0.56 ± 0.06) [23]. Even at 6 months postpartum—when the uterus has fully involuted, lactation-related fluid shifts have resolved, and normal menstrual cycles have typically resumed—At-Risk women maintained WHtR values significantly exceeding both the 0.50 metabolic threshold and the Low-Risk group mean (*p* < 0.001).

The 24-week postpartum anthropometric comparison (Table 2, Part B) demonstrates that the between-group WHtR difference widened slightly to 0.08 units by the end of the observation period, with the At-Risk group maintaining values 17% higher than the Low-Risk group. Descriptively, the Low-Risk group showed a steeper decline in waist and hip circumference that continued toward pre-pregnancy values, whereas the At-Risk group showed an initial decline by Week 4 followed by a plateau across weight, waist, and arm measurements. As reported below, however, this apparent difference in trajectory shape was not statistically supported: the group × time interaction was non-significant in the mixed-effects models, and the two strata are best described as running in parallel at different levels rather than diverging.

Linear mixed-effects models (random intercept per participant; all available observations) formally tested these patterns within each cohort. For WHtR, the effect of BMI stratum was significant in both Cohort A (*p* < 0.001) and Cohort B (*p* < 0.001), as was the effect of time (both *p* < 0.001), confirming that WHtR changed substantially across gestation and across the postpartum period. The group × time interaction, however, was not significant in either cohort (Cohort A: χ^2^(6) = 9.70, *p* = 0.14; Cohort B: χ^2^(6) = 6.57, *p* = 0.36), and the same pattern held for MUAC (Cohort A: χ^2^(6) = 9.35, *p* = 0.16; Cohort B: χ^2^(6) = 9.24, *p* = 0.16). The At-Risk stratum therefore maintained persistently higher central-adiposity indicators at every timepoint, with trajectories running broadly parallel to those of the Low-Risk stratum rather than diverging over time. Clinically, the separation remained meaningful at the end of follow-up: at 24 weeks postpartum the Low-Risk mean WHtR (0.484) was significantly below the 0.50 reference level (*p* = 0.013), whereas the At-Risk mean (0.558) remained significantly above it (*p* < 0.001). An attrition analysis found no significant difference between postpartum completers (n = 57) and non-completers (n = 25) in maternal age (*p* = 0.72), pre-pregnancy BMI (*p* = 0.60), baseline WHtR (*p* = 0.60), gravidity (*p* = 0.91), or BMI stratum (*p* = 1.00), supporting a missing-at-random assumption (Table S6). Full per-timepoint values and model results are provided in Supplementary Tables S1–S5.

## 4. Discussion

### 4.1. Principal Findings

In these two independent cohorts, women in the At-Risk stratum differed from those in the Low-Risk stratum on several anthropometric measures at both stages examined. At 12 weeks’ gestation, At-Risk women in Cohort A already presented with a mean WHtR of 0.58—above the 0.50 level commonly associated with elevated cardiometabolic risk—alongside higher MUAC, a pattern consistent with greater central and systemic adiposity before appreciable gestational weight gain. Because visceral fat was not measured directly, these anthropometric indicators are interpreted as surrogates rather than as confirmation of visceral adiposity. This initial disparity did not resolve postpartum; the At-Risk group plateaued above the risk threshold through 6 months postpartum, while their lower-BMI counterparts returned to safe anthropometric dimensions by Week 8. To contextualize the magnitude of the difference, a WHtR of 0.58 corresponds to a waist circumference exceeding the International Diabetes Federation’s ethnic-specific threshold for central obesity in Asian women (≥80 cm) [8].

The postpartum trajectory shapes are clinically important. The At-Risk trajectory in Cohort B flattened between Weeks 12 and 24. Because no metabolic, hormonal, or body-composition measurements were obtained, we cannot attribute this plateau to any specific physiological mechanism, such as set-point regulation; it is reported here as a descriptive anthropometric observation that would require a dedicated metabolic study to explain. The plateau was nonetheless apparent across central and peripheral measurements rather than at the waist alone. The concern that waist measurements in pregnancy merely reflect fetal growth is reduced, though not eliminated, by examining two timepoints when the fetal contribution is negligible (12 weeks’ gestation) or absent (24 weeks postpartum); at both, between-stratum anthropometric differences were evident. Because these timepoints fall in different women (Cohort A and Cohort B, respectively), they should be read as two independent snapshots rather than as serial confirmation within individuals.

### 4.2. Consistency with the International Evidence Base

The anthropometric patterns observed here are broadly consistent with adiposity data from Chinese, Indian, Korean, and Singaporean populations, in which cardiometabolic risk is documented at BMI levels below conventional Western thresholds [2,8,13]. They also align with evidence from predominantly European (Caucasian) cohorts, in which higher pre-pregnancy BMI and greater postpartum weight retention predict adverse cardiometabolic trajectories [11], and with longitudinal data showing that a greater pre-pregnancy-to-postpartum BMI change is associated with less favorable cardiometabolic risk factors [23]. This interpretation is reinforced by pooled evidence that adiposity measures capturing fat distribution, rather than BMI alone, track maternal risk. A meta-analysis of 11 cohort studies (27,675 women) found first- or second-trimester maternal central obesity associated with roughly 2.8-fold higher odds of gestational diabetes (adjusted OR 2.76, 95% CI 2.35–3.26), independent of BMI [24]. A larger systematic review of 70 studies (89,588 women) similarly reported that early-pregnancy waist circumference and related central-adiposity indicators, including waist-to-height ratio, were associated with higher odds of gestational diabetes and hypertensive disorders, and concluded that such measures may help direct antenatal care more efficiently than BMI alone [25]. Our study, however, measured anthropometric surrogates rather than the cardiometabolic outcomes examined in those cohorts; this concordance can inform, but does not by itself establish, the case for revisiting Asian maternal screening thresholds, which would require studies that link the ≥23 kg/m^2^ cut-off to clinical outcomes. Because these data describe the period before the 2022 Thai guideline took effect, they are best read as a historical baseline: they characterise central-adiposity patterns as they stood before the ≥23 kg/m^2^ threshold was embedded in national antenatal care, rather than as a call to revise a recommendation that is already in place.

### 4.3. Implications for Nursing Practice

The findings suggest possible directions for nursing practice across antenatal and postnatal care settings that would require confirmation before implementation. First, nurses and midwives could consider women with pre-pregnancy or early-pregnancy BMI ≥ 23 kg/m^2^ as a group that may warrant closer anthropometric assessment at the first antenatal booking visit, even when traditional WHO criteria classify them as normal weight. Integration of WHtR measurement (threshold ≥ 0.50) and MUAC into the routine first-booking assessment requires only a non-elastic tape, takes under 2 min, and is feasible in primary care and community health settings across Southeast Asia.

Second, identification of hidden obesity at 12 weeks gestation creates a critical and often missed window for nurse-led early nutritional counselling. At this timepoint, gestational weight gain is minimal, making elevated WHtR and MUAC unambiguous signals of pre-existing maternal adiposity. Nurse-led nutritional interventions in this setting typically combine individualized dietary assessment and goal-setting, culturally tailored advice on Thai meal composition and portion size, and structured follow-up. Programmes using the capability–opportunity–motivation–behavior (COM-B) framework—including a recent Thai nurse-led randomized controlled trial—have reported modest, potentially clinically relevant reductions in gestational weight gain [26]. Pregnancy constitutes a uniquely powerful teachable moment when women are highly motivated to adopt health-protective behaviours for their infant [27], and earlier identification may help make fuller use of this window.

Third, the postpartum findings in Cohort B are consistent with a possible need for extended postpartum nursing follow-up beyond the standard 6-week postnatal visit, which warrants formal evaluation. Women with pre-pregnancy BMI ≥ 23 kg/m^2^ who have not returned to WHtR < 0.50 by Week 12 postpartum should be referred to dietetic services or structured weight management support. A single postnatal check is insufficient to assess metabolic recovery in this population. Incorporating WHtR monitoring at Weeks 8, 12, and 24 into postnatal protocols represents a low-cost, evidence-based practice change that may position nurses to contribute to reducing intergenerational obesity risk.

### 4.4. Limitations

Several limitations materially constrain the inferences that can be drawn from these data. First, and most importantly, the pregnancy and postpartum data derive from two independent cohorts of different women; the design cannot show that individual women retain adiposity from pregnancy into the postpartum period, and the descriptive labels hidden obesity and sticky-fat should not be read as evidence of a within-woman process. Second, the strata were defined by BMI and then compared on anthropometric measures (waist and hip circumference, MUAC, and WHtR) that are themselves correlated with body size; larger values in the higher-BMI stratum are therefore expected to some degree and do not, on their own, validate BMI 23 kg/m^2^ as a metabolic threshold. Third, although linear mixed-effects models were fitted to the repeated measurements in each cohort and tested group, time, and group × time effects, the models could not be adjusted for covariates because the relevant variables were not captured in the parent dataset; the group effects reported are therefore unadjusted. Fourth, the two strata differed markedly in size (for example, 83 vs. 27 in the pregnancy cohort); although the primary WHtR comparison was well powered (post hoc power > 99%), a priori power calculations were not performed for the specific questions examined here, and the smaller subgroups limit precision and preclude reliable covariate adjustment. Fifth, and centrally, no metabolic or cardiometabolic measures of any kind were available: neither direct quantification of visceral adipose tissue by computed tomography or magnetic resonance imaging, nor fasting glucose, insulin, HbA1c, blood pressure, lipid profile, or clinical pregnancy outcomes. Validated anthropometric proxies (WHtR, MUAC) were used instead. Because this manuscript concerns central adiposity as a potential marker of cardiometabolic vulnerability, the absence of these biomarkers is a primary limitation: the study can characterise anthropometric patterns but cannot link them to metabolic risk in these women, and the BMI 23 kg/m^2^ cut-off is therefore not validated here as a metabolic threshold. Sixth, the postpartum cohort experienced 30.5% attrition by 24 weeks. Completers and non-completers did not differ significantly on any measured baseline characteristic (Table S6), and the mixed-effects models use all available observations rather than completers only, which mitigates this concern; nevertheless, unmeasured predictors of dropout cannot be excluded, and the small number of At-Risk completers (*n* = 23 at 24 weeks) limits the precision of the late postpartum estimates. Seventh, the dataset did not capture several established determinants of postpartum anthropometric recovery—breastfeeding status and duration, dietary intake, physical activity, gestational weight gain, parity, mode of delivery, socioeconomic status, and postpartum complications. Because these were neither measured nor adjusted for, the between-stratum differences reported here cannot be assumed to be independent of them, and no causal inference is warranted. Eighth, the eligible age range (18–45 years) spans a period over which resting metabolism and body composition change, which may add heterogeneity to BMI-based comparisons, although maternal age did not differ significantly between strata in either cohort. Finally, the study was conducted at a single tertiary referral centre in Northern Thailand in 2017–2018, so the findings may not generalize to rural or lower-resource settings or to other populations.

## 5. Conclusions

In two independent cohorts of Thai women, a pre-pregnancy BMI of ≥23 kg/m^2^ distinguished groups with markedly different anthropometric profiles across the maternal continuum. Women in the At-Risk stratum showed higher central adiposity indicators as early as 12 weeks’ gestation and, in the separate postpartum cohort, higher WHtR values that remained above the 0.50 level through 24 weeks. Because visceral fat and metabolic outcomes were not measured, and because the cohorts comprised different women, these findings are descriptive and do not, by themselves, validate BMI 23 kg/m^2^ as a metabolic threshold or establish within-woman fat retention. They do, however, add prospective anthropometric detail to the wider literature questioning reliance on the WHO 25 kg/m^2^ threshold in Asian maternal settings. Because the ≥23 kg/m^2^ threshold has since been adopted in Thai antenatal care (2022), these 2017–2018 data are best positioned as a pre-implementation baseline for future evaluation rather than as grounds for revising screening protocols, and they support the case for further, outcome-based research on the lower cut-off.

For nursing practice, the findings are hypothesis-generating rather than immediately prescriptive: they suggest that simple, low-cost anthropometric measures such as WHtR and MUAC could be evaluated as adjuncts at the first antenatal booking and during postpartum follow-up, particularly for women near the BMI 23 kg/m^2^ cut-off. Whether such screening—and extended postpartum follow-up beyond the single 6-week visit—improves outcomes should be tested in prospective, preferably within-woman, studies that link the cut-off to gestational weight gain and cardiometabolic outcomes in diverse Asian populations.

## Figures and Tables

**Table 1:** Demographic Characteristics of Pregnancy (cohort A) and postpartum (cohort B) Cohorts by BMI Risk-Group.

Characteristic	Pregnancy Cohort (*n* = 110)				Postpartum Cohort (*n* = 82)			
	Low-Risk (*n* = 83)	At-Risk (*n* = 27)	t	*p*	Low-Risk (*n* = 49)	At-Risk (*n* = 33)	t	*p*
Maternal age (years)	29.20 ± 5.39	28.30 ± 4.32	0.80	0.428	29.04 ± 5.46	29.11 ± 5.06	−0.06	0.950
Maternal height (cm)	157.49 ± 5.59	159.69 ± 5.82	−1.74	0.085	156.16 ± 6.23	157.88 ± 5.80	−1.26	0.213
Pre-pregnancy weight (kg)	49.64 ± 5.51	69.07 ± 13.35	−7.36	<0.001 *	49.28 ± 6.22	66.68 ± 11.34	−8.03	<0.001 *
Pre-pregnancy BMI (kg/m^2^)	20.00 ± 1.88	27.25 ± 5.47	−6.76	<0.001 *	20.16 ± 1.80	26.63 ± 3.43	−9.96	<0.001 *
Baseline MUAC (cm)	26.19 ± 2.42	32.52 ± 4.66	−6.58	<0.001 *	27.13 ± 2.14	31.61 ± 3.41	−6.70	<0.001 *
Baseline WHtR	0.49 ± 0.04	0.58 ± 0.08	−5.20	<0.001 *	0.60 ± 0.04	0.66 ± 0.04	−6.65	<0.001 *
Newborn birth weight (kg)	NA	NA	—	—	2.97 ± 0.46	3.00 ± 0.37	−0.28	0.777

Note. Data presented as Mean (SD). Low-Risk = BMI < 23.0 kg/m^2^; At-Risk = BMI ≥ 23.0 kg/m^2^; MUAC = Mid-Upper Arm Circumference; WHtR = Waist-to-Height Ratio; NA = not applicable. Measured at 12 weeks gestation (pregnancy cohort) and at delivery/Week 0 (postpartum cohort). * *p* < 0.001.

**Table 2:** Anthropometric Outcomes at 12 Weeks Gestation (Part A) and 24 Weeks Postpartum (Part B) by BMI Risk Group.

Variable	Low-Risk < 23 kg/m^2^ Mean (SD)	At-Risk ≥ 23 kg/m^2^ Mean (SD)	Mean Difference (95% CI)	t	*p*	Cohen’s d
Part A: Pregnancy Cohort Baseline (12 weeks gestation)|Low-Risk *n* = 83; At-Risk *n* = 27						
Waist circumference (cm)	77.43 ± 6.60	92.04 ± 11.28	14.61 (9.81, 19.41)	−6.20	<0.001 *	1.79
Hip circumference (cm)	92.51 ± 5.02	105.50 ± 9.83	12.99 (8.86, 17.12)	−6.42	<0.001 *	1.94
MUAC (cm)	26.19 ± 2.42	32.52 ± 4.66	6.33 (4.36, 8.29)	−6.58	<0.001 *	1.98
WHtR	0.49 ± 0.04	0.58 ± 0.08	0.09 (0.05, 0.12)	−5.20	<0.001 *	1.56
Part B: Postpartum Outcome (24 weeks postpartum)|Low-Risk *n* = 34; At-Risk *n* = 23						
Waist circumference (cm)	75.79 ± 6.18	88.30 ± 10.85	12.51 (7.42, 17.60)	−5.01	<0.001 *	1.49
Hip circumference (cm)	89.59 ± 5.39	99.94 ± 10.45	10.35 (5.51, 15.18)	−4.37	<0.001 *	1.32
MUAC (cm)	24.43 ± 2.91	28.80 ± 4.29	4.38 (2.30, 6.46)	−4.28	<0.001 *	1.24
WHtR	0.48 ± 0.04	0.56 ± 0.06	0.07 (0.04, 0.10)	−5.10	<0.001 *	1.52

Note. Low-Risk = BMI < 23 kg/m^2^; At-Risk = BMI ≥ 23 kg/m^2^; MUAC = Mid-Upper Arm Circumference; WHtR = Waist-to-Height Ratio; CI = confidence interval. * *p* < 0.001.

## Data Availability

The data supporting the findings of this study are available from the corresponding author upon reasonable request. Access to the full dataset is subject to ethical approval requirements in accordance with the conditions of the Institutional Review Board approvals (Faculty of Nursing CMU Code: FULL055-2017; Faculty of Medicine CMU Code: NONE-2017-05134; secondary analysis Code: 2569-EXEM001). The Python 3 analysis code for longitudinal trajectory visualization is also available upon reasonable request.

## Supplementary Materials

The following supporting information can be downloaded at: https://www.mdpi.com/article/10.3390/nursrep16080263/s1. Table S1: Longitudinal WHtR by pre-pregnancy BMI stratum—Cohort A (pregnancy); Table S2: Longitudinal WHtR by pre-pregnancy BMI stratum—Cohort B (postpartum); Table S3: Longitudinal MUAC (cm) by pre-pregnancy BMI stratum—Cohort A (pregnancy); Table S4: Longitudinal MUAC (cm) by pre-pregnancy BMI stratum—Cohort B (postpartum); Table S5: Linear mixed-effects models (random intercept per woman): group, time, and group × time effects within each cohort; Table S6: Attrition analysis—baseline comparison of completers vs non-completers (Cohort B); Table S7: STROBE Checklist—Cohort design [21]; Figure S1: Study Flow Diagram; Figure S2: Pregnancy anthropometric trajectories 12–36 weeks; Figure S3: Postpartum multi-panel recovery trajectories.

## Public Involvement Statement

No public involvement in any aspect of this research.

## Guidelines and Standards Statement

This study was prepared in accordance with the STROBE (Strengthening the Reporting of Observational Studies in Epidemiology) statement, which provides guidelines for the reporting of observational studies.

## Abbreviations

WHtR	Waist-to-height ratio
MUAC	Mid-upper arm circumference
BMI	Body mass index
COM-B	Capability–opportunity–motivation–behavior
DXA	Dual-energy X-ray absorptiometry
GWG	Gestational weight gain
ICC	Intraclass correlation coefficient
IDF	International Diabetes Federation
MoPH	Ministry of Public Health (Thailand)
WHO	World Health Organization
