# Supplementary material for: Central Adiposity Indicators by Pre-Pregnancy BMI at the 23 kg/m2 Cut-Off in Two Independent Cohorts of Thai Women During Pregnancy and Postpartum: A Descriptive Longitudinal Study
Source: Nurs Rep. 2026 Jul 29;16(8):263. doi: 10.3390/nursrep16080263 (PMC13516514; doi:10.3390/nursrep16080263)
Supplement: Supplementary file 1 [file nursrep-16-00263-s001.zip › Supplementary Figure.pdf]

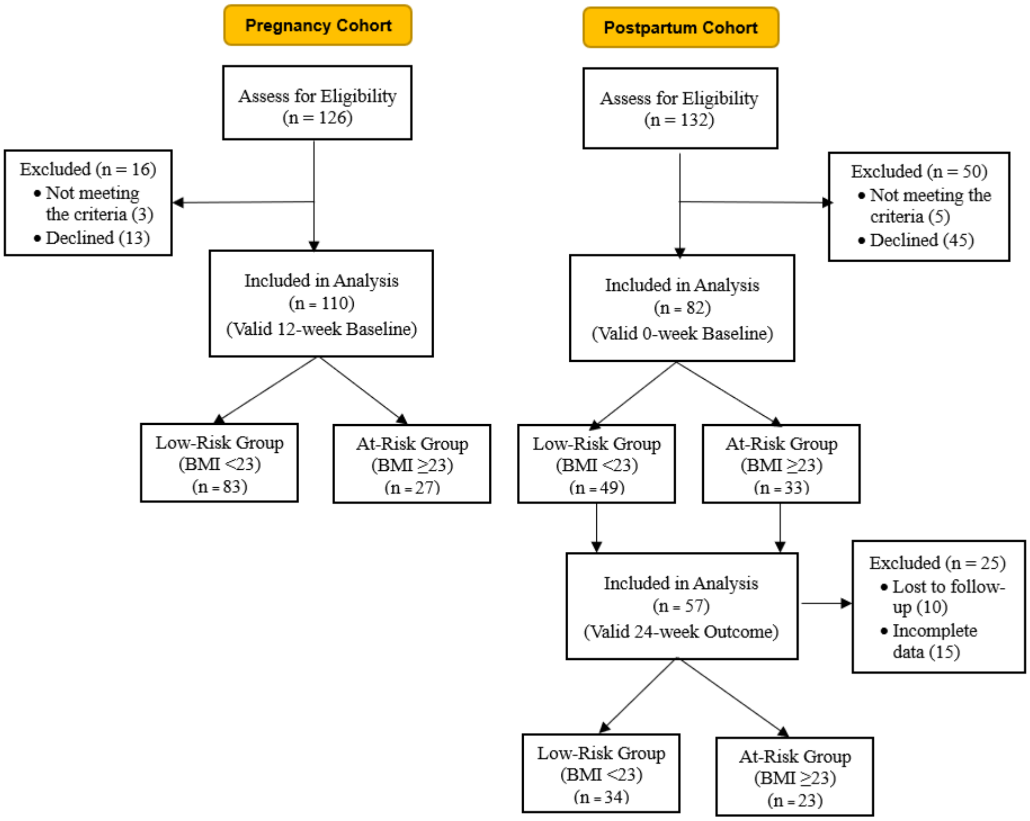

Figure S1. Study Flow Diagram

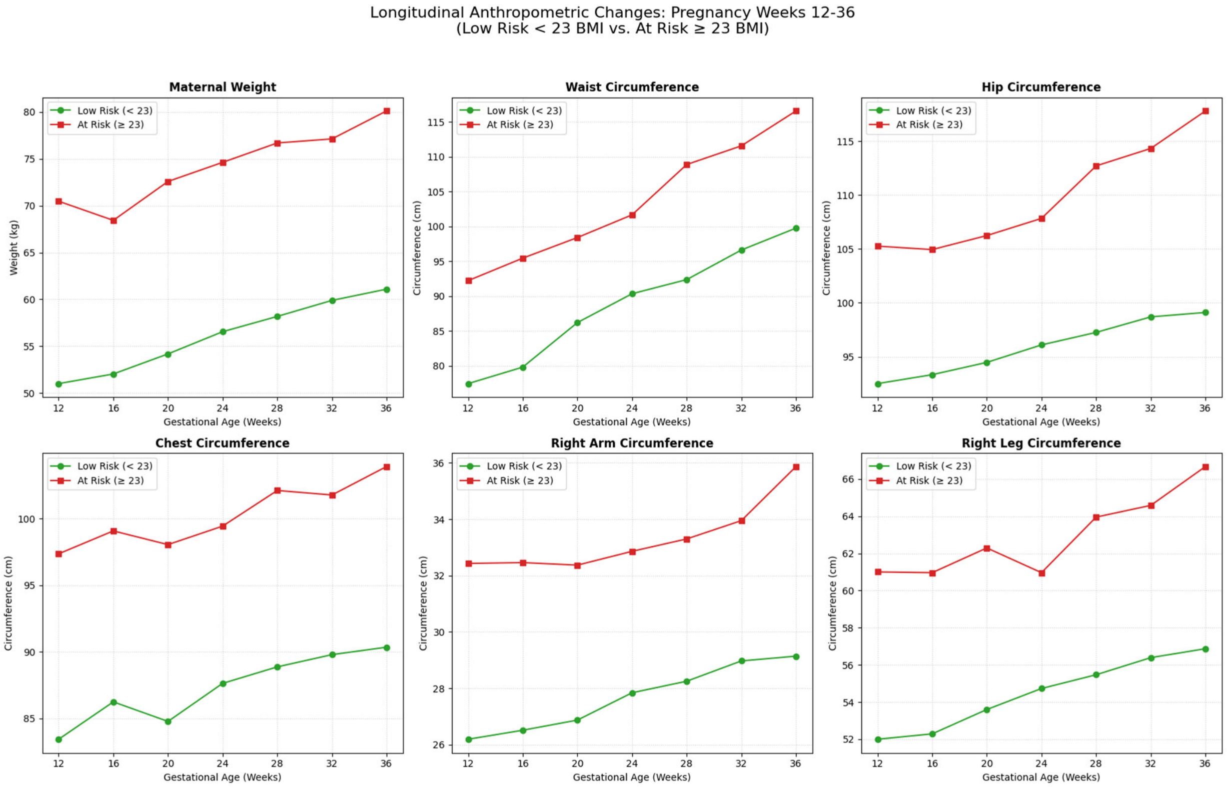

Figure S2. Pregnancy anthropometric trajectories 12–36 weeks

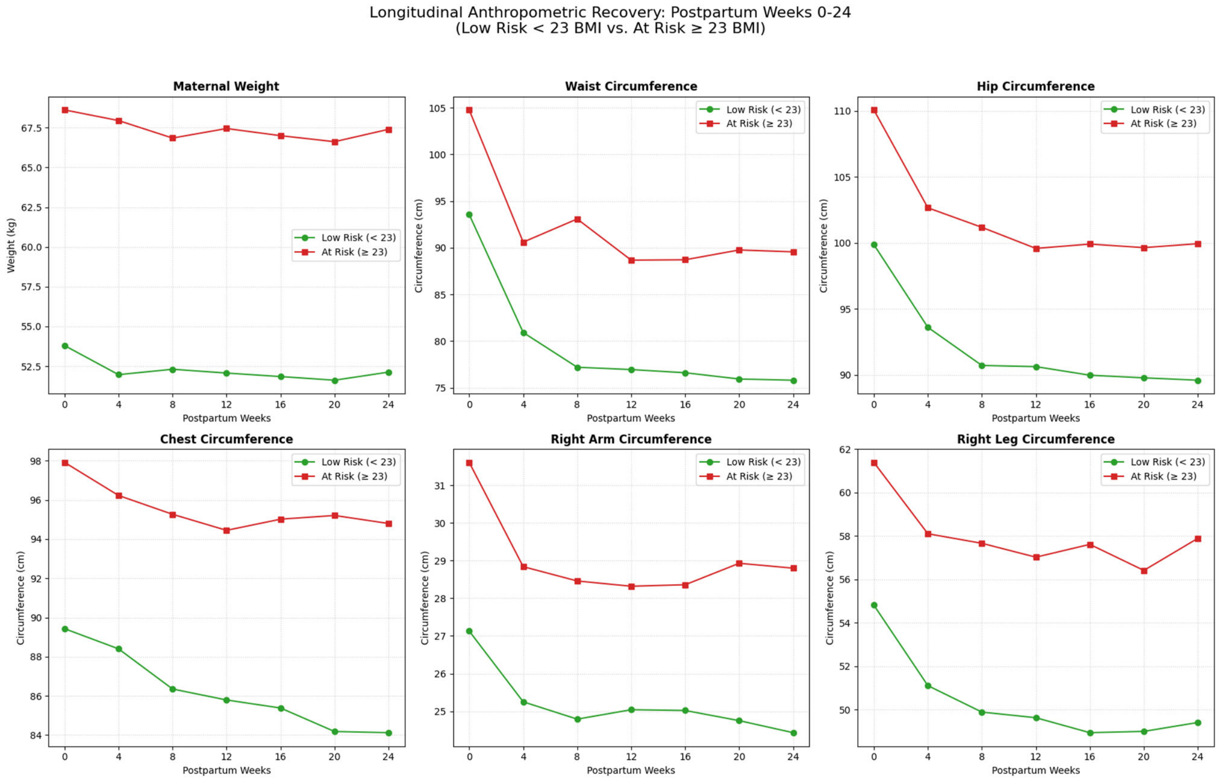

Figure S3. Postpartum multi-panel recovery trajectories
