# Supplementary material for: Central Adiposity Indicators by Pre-Pregnancy BMI at the 23 kg/m2 Cut-Off in Two Independent Cohorts of Thai Women During Pregnancy and Postpartum: A Descriptive Longitudinal Study
Source: Nurs Rep. 2026 Jul 29;16(8):263. doi: 10.3390/nursrep16080263 (PMC13516514; doi:10.3390/nursrep16080263)
Supplement: Supplementary file 1 [file nursrep-16-00263-s001.zip › Supplementary tables.pdf]

Supplementary Statistical Analysis

Additional analyses prepared in response to peer review (Reviewer 3): longitudinal descriptive statistics at every timepoint, linear mixed-effects models, and an attrition analysis. Manuscript nursrep-4396035.

A. Analytic notes. Cohort A (pregnancy) comprised 110 women (Low-Risk stratum, pre-pregnancy BMI <23 kg/m², n=83; At-Risk stratum, ≥23 kg/m², n=27). Cohort B (postpartum) comprised 82 women (Low-Risk n=49; At-Risk n=33). In both cohorts, women were stratified by true pre-pregnancy body mass index calculated from pre-pregnancy weight and height recorded in the antenatal medical record (weight/height²); no self-reported weights were used. Waist-to-height ratio (WHtR) was computed as waist circumference divided by height; mid-upper arm circumference (MUAC) denotes the right upper-arm circumference, matching the definition used in Table 2 of the manuscript. Between-group comparisons at each timepoint used the Mann–Whitney U test as a non-parametric sensitivity analysis, given the small strata and departure from normality in one group (Shapiro–Wilk p = 0.001 for the At-Risk stratum at 12 weeks' gestation). Welch's t-test is the primary test and is the one reported in Tables 1 and 2 of the main manuscript, since it yields the mean differences and effect sizes needed for clinical interpretation. The two tests agreed completely: across all 28 between-stratum comparisons they reached the same conclusion at α = 0.05, so no inference depends on the choice of test. Critically, the number of women contributing a valid measurement varies by timepoint and is smaller than the cohort total; every n below is the number actually analysed at that timepoint, and all statistics are computed on those women. Linear mixed-effects models used all available observations (maximum-likelihood estimation, random intercept per woman), which provides valid inference under a missing-at-random assumption and uses every measurement rather than only completers.

Table S1. Longitudinal WHtR by pre-pregnancy BMI stratum — Cohort A (pregnancy).

Week	Low-Risk n	Low mean	Low SD	At-Risk n	At-Risk mean	At-Risk SD	p*
**12**	67	0.492	0.044	25	0.578	0.079	<0.001
**16**	73	0.509	0.065	23	0.596	0.059	<0.001
**20**	67	0.551	0.037	22	0.613	0.061	<0.001
**24**	60	0.577	0.037	21	0.636	0.060	<0.001
**28**	58	0.590	0.069	20	0.681	0.076	<0.001
**32**	58	0.616	0.042	19	0.697	0.075	<0.001
**36**	49	0.637	0.039	15	0.729	0.090	<0.001

*Values are mean ± SD. n = women with a valid measurement at that gestational week. *Mann–Whitney U test, Low-Risk vs At-Risk.*

Table S2. Longitudinal WHtR by pre-pregnancy BMI stratum — Cohort B (postpartum).

Week	Low-Risk n	Low mean	Low SD	At-Risk n	At-Risk mean	At-Risk SD	p*
**0**	49	0.600	0.041	33	0.663	0.043	<0.001
**4**	45	0.519	0.037	31	0.590	0.050	<0.001
**8**	42	0.495	0.049	31	0.572	0.053	<0.001
**12**	42	0.494	0.033	30	0.558	0.055	<0.001
**16**	45	0.490	0.036	28	0.563	0.058	<0.001
**20**	42	0.487	0.039	27	0.568	0.066	<0.001
**24**	34	0.484	0.036	23	0.558	0.063	<0.001

*Values are mean ± SD. Week 0 = delivery. n = women with a valid measurement at that postpartum week. *Mann–Whitney U test.*

Table S3. Longitudinal MUAC (cm) by pre-pregnancy BMI stratum — Cohort A (pregnancy).

Week	Low-Risk n	Low mean	Low SD	At-Risk n	At-Risk mean	At-Risk SD	p*
**12**	67	26.194	2.418	26	32.519	4.664	<0.001
**16**	73	26.514	2.644	23	32.478	4.157	<0.001
**20**	67	26.864	2.522	22	32.386	4.421	<0.001
**24**	60	27.817	2.660	21	32.857	4.087	<0.001
**28**	58	28.219	2.966	20	33.300	3.672	<0.001
**32**	58	28.952	3.229	19	33.947	3.597	<0.001
**36**	49	29.124	3.573	15	35.867	5.055	<0.001

*Values are mean ± SD (cm). *Mann–Whitney U test.*

Table S4. Longitudinal MUAC (cm) by pre-pregnancy BMI stratum — Cohort B (postpartum).

Week	Low-Risk n	Low mean	Low SD	At-Risk n	At-Risk mean	At-Risk SD	p*
**0**	49	27.133	2.138	33	31.606	3.411	<0.001
**4**	46	25.246	2.704	31	28.839	2.882	<0.001
**8**	42	24.786	2.861	31	28.455	3.500	<0.001
**12**	42	25.036	2.630	30	28.317	3.986	<0.001
**16**	45	25.022	3.258	28	28.357	3.783	<0.001
**20**	42	24.750	2.859	28	28.929	3.887	<0.001
**24**	34	24.426	2.911	23	28.804	4.287	<0.001

*Values are mean ± SD (cm). *Mann–Whitney U test.*

Table S5. Linear mixed-effects models (random intercept per woman): group, time, and group × time effects within each cohort.

Cohort / outcome	Group (main effect)	Time (main effect)	Group × time interaction	Interpretation
**Cohort A — WHtR**	p <0.001	p <0.001	$\chi^2(6)$=9.70, p=0.138	Persistent group difference; trajectories essentially parallel (no significant interaction).
**Cohort A — MUAC**	p <0.001	p <0.001	$\chi^2(6)$=9.35, p=0.155	Persistent group difference; trajectories essentially parallel (no significant interaction).
**Cohort B — WHtR**	p <0.001	p <0.001	$\chi^2(6)$=6.57, p=0.362	Persistent group difference; trajectories essentially parallel (no significant interaction).
**Cohort B — MUAC**	p <0.001	p <0.001	$\chi^2(6)$=9.24, p=0.160	Persistent group difference; trajectories essentially parallel (no significant interaction).

Group main effect and time main effect were both statistically significant for every outcome in both cohorts (all p<0.001). The group × time interaction was non-significant in every case, indicating that the At-Risk stratum maintained persistently higher central-adiposity indicators across all timepoints with trajectories broadly parallel to the Low-Risk stratum, rather than diverging over time. Post hoc, at 24 weeks postpartum the Low-Risk stratum mean WHtR (0.484) was significantly below the 0.50 cardiometabolic reference level (one-sample p=0.013), whereas the At-Risk stratum mean (0.558) remained significantly above it (p<0.001).

Table S6. Attrition analysis — baseline comparison of completers vs non-completers (Cohort B).

Baseline characteristic	Completers (n=57)	Non-completers (n=25)	p
**Maternal age (years)**	28.93 ± 5.24	29.40 ± 5.45	0.717
**Pre-pregnancy BMI (kg/m^2)**	22.92 ± 4.23	22.43 ± 3.82	0.604
**Baseline WHtR (week 0)**	0.63 ± 0.05	0.62 ± 0.06	0.603
**Gravidity**	1.39 ± 0.49	1.40 ± 0.50	0.907
**At-Risk stratum, n (%)**	23 (40.4%)	10 (40.0%)	1.000

Completers and non-completers did not differ significantly on any measured baseline characteristic, including pre-pregnancy BMI stratum, supporting a missing-at-random assumption and mitigating concern about attrition bias. The mixed-effects models above additionally use all available data rather than completers only.

Note: WHtR trajectories for both cohorts are presented as Figure 1 of the main manuscript and are therefore not duplicated here. The per-timepoint values underlying that figure are given in Tables S1 and S2 above.

Table S7: STROBE Checklist — Cohort Study

Strengthening the Reporting of Observational Studies in Epidemiology

von Elm E, et al. Lancet 2007;370:1453–1457. doi:10.1016/S0140-6736(07)61602-X

Manuscript: Central Adiposity Indicators by Pre-Pregnancy BMI at the 23 kg/m² Cut-Off in Two Independent Cohorts of Thai Women During Pregnancy and Postpartum: A Descriptive Longitudinal Study

Authors: Piyanut Xuto, Piyaporn Prasitwattanaseree, Lawitra Khiaokham, Daniel Bressington, Patompong Khaw-on

Target Journal: Nursing Reports (MDPI) | Study Design: Descriptive Longitudinal Dual-Cohort, Independent Cohorts (Observational — Cohort)

Item No.	Recommendation	Page / Section	Authors' Response (Location in Manuscript)
**TITLE AND ABSTRACT**			
**1(a)**	Indicate the study's design with a commonly used term in the title or abstract.	Title; Abstract	The abstract states 'descriptive longitudinal dual-cohort study' and specifies that the two cohorts are independent, with no woman contributing to both. The title does not include a design label; design is clearly identified in the Abstract (Methods line) and in Section 2.1.
**1(b)**	Provide in the abstract an informative and balanced summary of what was done and what was found.	Abstract (p. 1)	Structured abstract provided with Objectives, Methods (design, setting, participants, outcomes, timepoints, validation), Results (key WHtR and MUAC findings with 95% CI, Cohen's d), and Conclusions (two-phenotype summary and nursing practice implications).
**INTRODUCTION**			
**2**	Explain the scientific background and rationale for the investigation being reported.	Section 1 (pp. 2–3)	Background covers: global obesity epidemic and maternal health consequences; WHO BMI threshold derived from Caucasian data; Asian paradox/thin-fat phenotype; visceral adipose tissue mechanisms; WHO Expert Consultation and 10-country Southeast Asian consensus endorsing BMI ≥23 kg/m²; Thai MoPH 2022 antenatal care policy; evidence gap for longitudinal maternal anthropometric validation.
**3**	State specific objectives, including	Section 1 (p. 3)	Two descriptive hypotheses stated explicitly, framed as group-level anthropometric

Item No.	Recommendation	Page / Section	Authors' Response (Location in Manuscript)
	any prespecified hypotheses.		comparisons rather than causal claims: (a) hidden obesity — higher central adiposity indicators detectable at 12 weeks gestation, before appreciable gestational weight gain; (b) sticky-fat — higher and more slowly declining WHtR through 24 weeks postpartum, beyond the uterine involution window, observed in a separate cohort of different women. The manuscript explicitly states that because the two cohorts comprise different women, these patterns represent group-level differences rather than demonstrated within-woman fat retention.
**METHODS**			
**4**	Study design: Present key elements of study design early in the paper.	Section 2.1 (p. 3-4)	Described as a 'prospective longitudinal study employing a dual-cohort design'; explicitly states that Cohort A (pregnancy) and Cohort B (postpartum) comprise different women recruited as separate samples, with no participant in both. Secondary analysis of data collected October 2017 – August 2018 at Maharaj Nakorn Chiang Mai Hospital. Parent study (Z-Size Ladies project) described with reference [20].
**5**	Setting: Describe the setting, locations, and relevant dates, including periods of recruitment, exposure, follow-up, and data collection.	Section 2.1 (p. 3)	Setting: tertiary referral obstetric center, Maharaj Nakorn Chiang Mai Hospital, Northern Thailand. Dates: October 2017 – August 2018. Cohort A: 7 gestational timepoints (12–36 weeks). Cohort B: 7 postpartum timepoints (Weeks 0–24, i.e., delivery to 6 months postpartum).
**6**	Participants: (a) Cohort study — Give the eligibility criteria, and the sources and methods of selection. Describe methods of follow-up. (b) For matched studies, give matching criteria and number of exposed and	Section 2.3 (p. 3)	Eligibility: Thai women aged 18–45 years, singleton pregnancy, written consent. Exclusion: fetal anomaly, pre-existing DM or hypertension, medications affecting body composition (corticosteroids/antipsychotics), physical disability. Stratification by pre-pregnancy BMI: Low-Risk (<23 kg/m²) vs. At-

Item No.	Recommendation	Page / Section	Authors' Response (Location in Manuscript)
	unexposed.		Risk (≥23 kg/m²). Selection from antenatal/postpartum attendees at study site. Follow-up: Cohort A — 7 gestational timepoints; Cohort B — 7 postpartum timepoints through 24 weeks.
**7**	Variables: Clearly define all outcomes, exposures, predictors, potential confounders, and effect modifiers. Give diagnostic criteria, if applicable.	Sections 2.2–2.4 (pp. 3–4)	Primary outcome: WHtR (waist circumference ÷ height; risk threshold ≥0.50). Secondary outcome: MUAC. Exposure/predictor: pre-pregnancy BMI stratified at Asian-specific threshold (23 kg/m²). Additional measures: waist, hip, chest, leg circumferences; height; weight. Potential confounders (breastfeeding, physical activity, diet, gestational weight gain, parity, mode of delivery, socioeconomic status, postpartum complications) noted as limitations (Section 4.4).
**8**	Data sources/measurement: For each variable of interest, give sources of data and details of methods of assessment (measurement). Describe comparability of assessment methods if there is more than one group.	Sections 2.4–2.5 (pp. 3–4)	Height: Seca 206 stadiometer (Seca, Hamburg, Germany; 0.1 cm). Weight: Tanita BC-418 digital scale (Tanita, Tokyo, Japan; 0.1 kg). BMI: calculated (kg/m²). Pre-pregnancy weight: obtained from the antenatal medical record for all participants in both cohorts; no self-reported weights were used (0% self-report). Circumferences: standardized protocols from parent study [20], triplicate at each timepoint. Validation: Size Stream SS20 3D scanner (n = 45; r = 0.92–0.99). Self-measurement ICC >0.95 (n = 30).
**9**	Bias: Describe any efforts to address potential sources of bias.	Sections 2.3, 2.5, 4.4	Measurement bias minimized: trained research nurses, standardized protocols, triplicate measurements, private room at controlled temperature, bladder voided before measurement. Validated against 3D body scanner. 12-week baseline timing selected to minimize fetal contribution to abdominal girth. Attrition bias acknowledged in Section 4.4 (30.5% by Week 24); noted as limitation.
**10**	Study size: Explain how the study	Sections 2.3,	Secondary analysis of parent study (Z-Size

Item No.	Recommendation	Page / Section	Authors' Response (Location in Manuscript)
	size was arrived at.	4.4 (pp. 4, 11-12)	Ladies); a priori sample size calculation not performed for current hypotheses. Post hoc power analysis confirmed >99% power for primary WHtR outcome. Limitation acknowledged. Final analyzed sample: Cohort A n = 110 (83 Low-Risk, 27 At-Risk); Cohort B n = 82 at baseline, n = 57 at 24 weeks.
**11**	Quantitative variables: Explain how quantitative variables were handled in the analyses. If applicable, describe which groupings were chosen and why.	Sections 2.3, 2.6 (pp. 4, 5-6)	BMI dichotomized at 23 kg/m² (Low-Risk vs. At-Risk) based on WHO Expert Consultation and Southeast Asian obesity consensus threshold. Rationale stated: direct test of clinical utility of the threshold; multiple subgroups would reduce power and clinical interpretability. Continuous anthropometric outcomes analyzed as means (SD); differences reported with 95% CI and Cohen's d.
**12(a)**	Statistical methods: Describe all statistical methods, including those used to control for confounding.	Section 2.6 (p. 5,6)	Data analyzed using SPSS Version 20.0 (IBM Corporation, Armonk, NY, USA). Welch's t-test (unequal variances) for between-stratum comparisons at each pre-specified timepoint; Cohen's d for effect size. Two pre-registered primary outcomes (WHtR at 12 weeks gestation; WHtR at 24 weeks postpartum) tested in a confirmatory framework, so Bonferroni correction was not applied; serial timepoint p-values reported descriptively for trajectory characterization. Because each cohort contributes repeated measurements on the same women, linear mixed-effects models (random intercept per participant, maximum-likelihood estimation; fixed effects for group, time, and group × time) were additionally fitted separately in each cohort for WHtR and MUAC. Between-stratum comparisons at individual timepoints used the Mann–Whitney U test given small strata (full results in Supplementary Tables S1–S4; mixed-model results in Table S5). An attrition analysis compared baseline characteristics of postpartum completers and non-completers (Table S6).

Item No.	Recommendation	Page / Section	Authors' Response (Location in Manuscript)
**12(b)**	Describe any methods used to examine subgroups and interactions.	Section 4.4 (p. 8)	Subgroup analyses by parity, mode of delivery, or breastfeeding status not performed due to insufficient power; explicitly stated as a limitation.
**12(c)**	Explain how missing data were addressed.	Section 3.1 (p. 5)	Cohort B: 25 participants (30.5%) lost to follow-up by Week 24. Table 1 presents baseline characteristics for all 82 enrolled postpartum participants; 24-week outcomes reported for 57 completers. Complete-case analysis employed for 24-week endpoint.
**12(d)**	Cohort study: If applicable, explain how loss to follow-up was addressed.	Sections 3.1, 4.4	30.5% attrition acknowledged. Baseline characteristics of all 82 enrollees retained in Table 1. Potential differential dropout noted as limitation (Section 4.4). Attrition analysis by BMI group is identified as a study limitation.
**12(e)**	Describe any sensitivity analyses.	Not applicable	No formal sensitivity analyses conducted. Post hoc power calculation reported (>99%). Limitation acknowledged regarding absence of subgroup and sensitivity analyses.
**RESULTS**			
**13(a)**	Report numbers of individuals at each stage of study — e.g., numbers potentially eligible, examined for eligibility, confirmed eligible, included in the study, completing follow-up, and analysed.	Section 3.1 (p. 6)	Cohort A: 126 assessed → 16 excluded (3 ineligible, 13 declined) → 110 analyzed. Cohort B: 132 assessed → 50 excluded (5 ineligible, 45 declined) → 82 enrolled → 57 completed 24 weeks (attrition n = 25, 30.5%). STROBE flow diagram: Supplementary Figure S1.
**13(b)**	Give reasons for non-participation at each stage.	Section 3.1 (p. 6)	Cohort A: 3 did not meet inclusion criteria; 13 declined participations. Cohort B: 5 did not meet inclusion criteria; 45 declined participations. Reasons for 24-week attrition not specified (noted as limitation).
**13(c)**	Consider use of a flow diagram.	Supplementary Figure S1	STROBE participant flow diagram provided as Supplementary Figure S1 (uploaded as supplementary file).

Item No.	Recommendation	Page / Section	Authors' Response (Location in Manuscript)
**14(a)**	Give characteristics of study participants (e.g., demographic, clinical, social) and information on exposures and potential confounders.	Section 3.1; Table 1 (p. 6-7)	Table 1 presents maternal age, height, pre-pregnancy weight, pre-pregnancy BMI, baseline MUAC, baseline WHtR, and newborn birth weight (Cohort B) by BMI risk group for both cohorts. Mean (SD) and independent-samples t-test results reported.
**14(b)**	Indicate number of participants with missing data for each variable of interest.	Section 3.1 (p. 6)	Missing data not reported at item level. Overall attrition of 25/82 (30.5%) in Cohort B by Week 24 is reported. Missing data at individual measurement timepoints not quantified separately.
**14(c)**	Cohort study: Summarise follow-up time (e.g., mean and total amount of follow-up).	Section 2.2 (p. 4); Figure S2-S3	Cohort A: 7 gestational timepoints from 12 to 36 weeks (24-week observation window). Cohort B: 7 postpartum timepoints from Week 0 (delivery) to Week 24 (6 months postpartum).
**15**	Cohort study: Report numbers of outcome events or summary measures over time.	Sections 3.3–3.4; Table 2 (p. 7)	Primary outcomes (WHtR) reported at 12 weeks gestation (Table 2, Part A) and 24 weeks postpartum (Table 2, Part B) with mean (SD), mean difference, 95% CI, t, p, and Cohen's d. Longitudinal trajectories described in text (Sections 3.3–3.4) and Figure 1 (WHtR) and Supplementary Figure S2 (all measures).
**16(a)**	Give unadjusted estimates and, if applicable, confounder-adjusted estimates and their precision (e.g., 95% confidence interval). Make clear which confounders were adjusted for and why.	Sections 3.3–3.4; Table 2	Unadjusted between-group differences reported with 95% CI, t-statistics, p-values, and Cohen's d at each timepoint. No confounder-adjusted estimates calculated; demographic comparability confirmed (age and height non-significant, Table 1). Unadjusted analysis justified by design.
**16(b)**	Report category boundaries when continuous variables were categorized.	Section 2.3 (p. 4)	BMI dichotomized at 23 kg/m²: Low-Risk (<23 kg/m²) and At-Risk (≥23 kg/m²), consistent with WHO Expert Consultation and 10-country Southeast Asian obesity consensus threshold. WHtR risk threshold of 0.50 stated and referenced [14–16].

Item No.	Recommendation	Page / Section	Authors' Response (Location in Manuscript)
**16(c)**	If relevant, consider translating estimates of relative risk into absolute risk for a meaningful time period.	Section 4.1 (p. 9-10)	WHtR values contextualized against the 0.50 metabolic risk threshold and the IDF ethnic-specific central obesity threshold (waist ≥80 cm in Asian women). Percentage difference at 24 weeks stated (17% higher in At-Risk group).
**17**	Report other analyses done — e.g., analyses of subgroups and interactions, and sensitivity analyses.	Sections 2.6, 4.4	No subgroup analyses (parity, delivery mode, breastfeeding) performed, acknowledged as a limitation. Within-cohort longitudinal trajectories tested with linear mixed-effects models (group, time, and group × time effects; Table S5); group × time interaction was non-significant for WHtR and MUAC in both cohorts, indicating persistently parallel rather than diverging trajectories. An attrition analysis compared postpartum completers and non-completers on baseline characteristics, finding no significant differences (Table S6), supporting a missing-at-random assumption. Post hoc power calculation reported (>99% for primary WHtR outcome).
**DISCUSSION**			
**18**	Summarize key results with reference to study objectives.	Section 4.1 (p. 9-10)	Principal findings section summarizes: (1) WHtR = 0.58 in At-Risk women at 12 weeks gestation (exceeding the 0.50 threshold), a pattern consistent with the descriptively labeled hidden obesity pattern; (2) in the separate postpartum cohort, the At-Risk group maintained WHtR >0.56 through 24 weeks postpartum while the Low-Risk group returned to WHtR <0.50 by Week 8, a pattern consistent with the descriptively labeled sticky-fat pattern. Both findings are explicitly framed as group-level anthropometric differences between independent cohorts, not as confirmed within-woman fat retention.
**19**	Discuss limitations of the study, taking into account sources of potential bias or imprecision.	Section 4.4 (p. 11-12)	Nine limitations discussed: (1) the pregnancy and postpartum data derive from two independent cohorts of different women, so the

Item No.	Recommendation	Page / Section	Authors' Response (Location in Manuscript)
	Discuss both direction and magnitude of any potential bias.		design cannot show within-woman fat retention, and the descriptive labels hidden obesity and sticky-fat should not be read as evidence of a within-woman process; (2) strata were defined by BMI and then compared on anthropometric measures correlated with body size, so larger values in the higher-BMI stratum are expected to some degree; (3) linear mixed-effects models could not be adjusted for covariates absent from the parent dataset, so group effects are unadjusted; (4) markedly unequal strata sizes (e.g., 83 vs. 27 in the pregnancy cohort) limit precision and preclude covariate adjustment, though the primary WHtR comparison was well powered post hoc (>99%; a priori power calculation not performed); (5) no metabolic or cardiometabolic measures of any kind were available (no DXA/CT/MRI visceral fat imaging, fasting glucose, insulin, HbA1c, blood pressure, lipid profile, or clinical pregnancy outcomes), so the BMI 23 kg/m² cut-off is not validated here as a metabolic threshold; (6) 30.5% attrition in Cohort B by 24 weeks, though completers and non-completers did not differ significantly on measured baseline characteristics (Table S6); (7) no data on breastfeeding status/duration, diet, physical activity, gestational weight gain, parity, mode of delivery, socioeconomic status, or postpartum complications; (8) the eligible age range (18–45 years) may add heterogeneity, though maternal age did not differ significantly between strata; (9) single tertiary centre in Northern Thailand, 2017–2018 — limited generalizability to rural/lower-resource settings or other populations.
**20**	Give a cautious overall interpretation of results considering objectives, limitations, multiplicity of analyses, and results from similar studies.	Sections 4.2, 5 (pp. 10, 12)	Findings interpreted as consistent with adiposity data from Chinese, Indian, Korean, and Singaporean populations (Section 4.2), while explicitly noting the study measured anthropometric surrogates rather than the cardiometabolic outcomes examined in those

Item No.	Recommendation	Page / Section	Authors' Response (Location in Manuscript)
			cohorts. Because the data predate Thailand's 2022 antenatal adoption of the ≥23 kg/m² threshold, they are framed as a historical, pre-implementation baseline rather than a call to revise a recommendation already in place. Conclusions framed with cautious, hedged language ('add prospective anthropometric detail', 'hypothesis-generating rather than immediately prescriptive'); do not claim to validate the BMI 23 kg/m² cut-off as a metabolic threshold. Future research direction (within-woman, outcome-based studies) stated (Section 5).
**21**	Discuss the generalisability (external validity) of the study results.	Sections 4.2, 4.4 (pp. 10-11)	Cross-regional consistency with other Asian populations discussed (Section 4.2). Single-centre limitation and Northern Thailand setting acknowledged as constraints on generalizability (Section 4.4). Implications framed for Southeast Asian health systems broadly.
**OTHER INFORMATION**			
**22**	Give the source of funding and the role of the funders for the present study and, if applicable, for the original study on which the present article is based.	Funding section (p. 13)	Funding: NECTEC [Grant No. JRA-NT-2560-5438-TH] and CMU International Visiting Professor Fellowship. Role of funders: 'no role in the design of the study; in the collection, analyses, or interpretation of data; in the writing of the manuscript; or in the decision to submit.'

Note: This checklist applies STROBE Cohort Study criteria. The dual-cohort design (Cohort A: pregnancy; Cohort B: postpartum) constitutes two prospective observational cohorts with independent recruitment, serial anthropometric follow-up, and pre-specified primary outcomes. Items not applicable to this study are annotated accordingly.
